# VS-4718 Antagonizes Multidrug Resistance in ABCB1- and ABCG2-Overexpressing Cancer Cells by Inhibiting the Efflux Function of ABC Transporters

**DOI:** 10.3389/fphar.2018.01236

**Published:** 2018-10-30

**Authors:** Ning Ji, Yuqi Yang, Chao-Yun Cai, Zi-Ning Lei, Jing-Quan Wang, Pranav Gupta, Qiu-Xu Teng, Zhe-Sheng Chen, Dexin Kong, Dong-Hua Yang

**Affiliations:** ^1^Tianjin Key Laboratory on Technologies Enabling Development of Clinical Therapeutics and Diagnostics, School of Pharmacy, Tianjin Medical University, Tianjin, China; ^2^Department of Pharmaceutical Sciences, College of Pharmacy and Health Sciences, St. John's University, Queens, NY, United States

**Keywords:** VS-4718, multidrug resistance (MDR), ATP-binding cassette (ABC) transporter, P-glycoprotein (P-gp/ABCB1), breast cancer resistance protein (BCRP/ABCG2)

## Abstract

Overexpression of ATP-binding cassette (ABC) transporters is one of the most important mechanisms responsible for multi-drug resistance (MDR). VS-4718, a tyrosine kinase inhibitor targeting focal adhesion kinase (FAK) with a potential anticancer effect, is currently evaluated in clinical trials. In this study, we investigated whether VS-4718 could reverse MDR mediated by ABC transporters, including ABCB1, ABCG2, and ABCC1. The results showed that VS-4718 significantly reversed ABCB1- and ABCG2-mediated MDR, but not MDR mediated by ABCC1. Treatment of VS-4718 did not alter the protein level and subcellular localization of ABCB1 or ABCG2. Mechanism studies indicated that the reversal effects of VS-4718 were related to attenuation of the efflux activity of ABCB1 and ABCG2 transporters. ATPase analysis indicated that VS-4718 stimulated the ATPase activity of ABCB1 and ABCG2. Docking study showed that VS-4718 interacted with the substrate-binding sites of both ABCB1 and ABCG2, suggesting that VS-4718 may affect the activity of ABCB1 and ABCG2 competitively. This study provided a novel insight for MDR cancer treatment. It indicated that combination of VS-4718 with antineoplastic drugs could attenuate MDR mediated by ABCB1 or ABCG2 in ABCB1- or ABCG2-overexpressing cancer cells.

## Introduction

Multidrug resistance (MDR) remains a major challenge that contributes to the failure of cancer chemotherapy (Szakács et al., 2006; Kartal-Yandim et al., 2016). MDR in cancer leads to synchronous resistance of cancer cells to structurally unrelated anticancer drugs. Several mechanisms contribute to cancer MDR, including reduced apoptosis, advanced DNA damage repair mechanisms, or altered drug metabolism. However, ATP binding cassette (ABC) transporters play a critical role in inducing MDR in cancer cells (Gottesman et al., 2002; Eckford and Sharom, 2009).

The ABC transporters contain diverse groups of active membrane transporters with important physiological and pharmacological roles (Dassa and Bouige, 2001). Divided into 7 subfamilies from ABCA to ABCG, the human ABC protein family has 49 ABC proteins and 48 of them have functions (Stavrovskaya and Stromskaya, 2008; Eckford and Sharom, 2009). Collectively, they are widely expressed in the placenta, blood brain barrier (BBB), intestines, liver, and kidneys to restrict the bioavailability of administered drugs (Linton, 2007; Linton and Higgins, 2007), transporting and regulating levels of physiological substrates such as lipids, porphyrins, and sterols (Wu and Ambudkar, 2014). The ABC transporters also play an important role in MDR, especially the ABC transporter subfamily B member 1 (ABCB1/P-glycoprotein, P-gp) and-subfamily G member 2 (ABCG2/breast cancer resistance protein, BCRP). The ABC transporters significantly decrease the intracellular concentration of certain anticancer drugs by pumping substrate drugs out of cancer cells, which becomes a major impediment to chemotherapy. It is well documented that the expression of ABC transporters are associated with the level of response of chemotherapy and the progression of malignancy (Liu et al., 2013, 2014; Ali and Elsalakawy, 2014; Xie et al., 2014; Yang et al., 2015). Thus, inhibiting the efflux function of ABC transporters is of great importance to enhance the efficacy of chemotherapy (Shukla et al., 2008).

Previously, we found that some tyrosine kinase inhibitors (TKIs) could attenuate ABC transporter-mediated MDR. For example, dacomitinib, an epidermal growth factor receptor (EGFR) inhibitor, directly inhibits the efflux activity of ABCB1 and ABCG2, thereby decreasing the efflux of certain anticancer drugs and subsequently increases their intracellular accumulation, finally reversing the MDR (Zhang et al., 2018).

VS-4718 (PND-1186) is a selective focal adhesion kinase (FAK) inhibitor with potential anti-cancer activity in breast cancer and ovarian cancer (Tanjoni et al., 2010; Tancioni et al., 2014). It is currently evaluated in clinical trials (NCT02215629, NCT01849744, and NCT02651727). Recent studies have shown that VS-4718 could drive depletion of regulatory T cells (Tregs) and promotes CD8^+^ T cell-mediated anti-tumor response (Serrels et al., 2015). However, there is hardly any research indicating the sensitizing effects of VS-4718 in ABC transporter-overexpressing cancer cells. Here we report for the first time that VS-4718 shows a significant effect on reversing ABCB1- and ABCG2-mediated MDR at non-toxic concentrations.

## Materials and methods

### Chemicals

VS-4718 (PND-1186) was a gift from Chemie Tek (Indianapolis, IN). Bovine serum albumin (BSA), fetal bovine serum (FBS), Dulbecco's modified Eagle's Medium (DMEM), penicillin/streptomycin and 0.25% trypsin were purchased from Corning Incorporated (Corning, NY). The monoclonal antibodies for ABCG2 (catalog number MAB4146, Lot number 3026758, clone BXP-21) were purchased from Millipore (Billerica, MA). Paclitaxel, doxorubicin, cisplatin, vincristine, mitoxantrone, verapamil, the monoclonal antibodies for ABCB1 (catalog number P7965, Lot number 067M4761V, clone F4), dimethylsulfoxide (DMSO), 3-(4,5-dimethylthiazol-yl)-2,5-diphenyltetrazolium bromide (MTT), Triton X-100, 4',6-diamidino-2-phenylindole (DAPI), and paraformaldehyde, were purchased from Sigma-Aldrich (St. Louis, MO). The monoclonal antibody for GAPDH (catalog number MA5-15738, Lot number SA247966, clone GA1R), Alexa Fluor 488 conjugated goat anti-mouse IgG secondary antibody, SN-38 and MK571, were purchased from Thermo Fisher Scientific Inc (Rockford, IL). HRP-conjugated rabbit anti-mouse IgG secondary antibody (catalog number 7076S, Lot number 32) were purchased from Cell Signaling Technology Inc (Danvers, MA). Ko143 was a product from Enzo Life Sciences (Farmingdale, NY). [^3^H]-paclitaxel (15 Ci/mmol) and [^3^H]-mitoxantrone (2.5 Ci/mmol) were purchased from Moravek Biochemicals, Inc., (Brea, CA). All other chemicals were purchased from Sigma Chemical Co (St. Louis, MO).

### Cell lines and cell culture

The ABCB1-overexpressing resistant KB-C2 cells were established by step-wise increased concentration of colchicine to parental human epidermoid carcinoma KB-3-1 cells and were cultured in medium with 2 μg/mL colchicine (Akiyama et al., 1985). The ABCC1-overexpressing KB-CV60 cells were cloned from KB-3-1 cells and were maintained in medium with 1 μg/mL cepharanthine and 60 ng/mL vincristine (Taguchi et al., 1997). Both KB-C2 and KB-CV60 and their parental KB-3-1 cells were kindly provided by Dr. Shin-ichi Akiyama (Kagoshima University, Kagoshima, Japan). The human colon cancer SW620 cells and the doxorubicin-selected ABCB1-overexpressing resistant subline SW620/Ad300 were used for ABCB1 reversal study and the SW620/Ad300 cells were cultured in medium with 300 ng/mL doxorubicin (Bates et al., 1993). The human non-small cell lung cancer (NSCLC) NCI-H460 cells and the mitoxantrone-selected subline ABCG2-overexpressing NCI-H460/MX20 cells were used for ABCG2 reversal study and the NCI-H460/MX20 cells were maintained in medium with 20 ng/mL mitoxantrone (Robey et al., 2001). The human colon carcinoma cell line S1 and its mitoxantrone-selected derivative ABCG2 overexpressing S1-M1-80 cells were used for ABCG2 reversal study and the S1-M1-80 cells were maintained in the medium with 80 μM mitoxantrone (Miyake et al., 1999). HEK293/pcDNA3.1 and HEK293/ABCB1 were established by transfecting the human embryonic kidney HEK293 cells with empty and ABCB1 expressing vector respectively (Fung et al., 2014). HEK293/pcDNA3.1 and HEK293/ABCG2 were transfected with either an empty vector pcDNA3.1 or a pcDNA3.1 vector containing a full length ABCG2. Transfected cells were selected with complete culture medium containing G418 (2 mg/ml). SW620 and SW620/Ad300, NCI-H460 and NCI-H460/MX20,S1 and S1-M1-80, HEK293/ABCG2 were kindly provided by Drs. Susan Bates and Robert Robey (NCI, NIH, Bethesda, MD). HEK293/ABCB1 were kindly provided by Dr. Suresh V. Ambudkar (NCI, NIH, Bethesda, MD). Each aforementioned cell line was cultured in DMEM medium containing 10% fetal bovine serum and 1% penicillin/streptomycin at 37°C in a humidified atmosphere containing 5% CO_2_. All cells were grown as an adherent monolayer and drug-resistant cells were grown in drug-free culture media for more than 2 weeks before assay. All cells were tested by DAPI staining and found free of mycoplasma contamination before being used for experiments.

### Cell viability and reversal experiments

Cell viability and reversal fold were determined using MTT assay as previously described (Zhang X. Y. et al., 2016). Briefly, for the reversal study, each type of cells were harvested and resuspended, and seeded evenly onto a 96-well plate at a final concentration of 5 × 10^3^ cells per well in 160 μl medium. After incubating for 24 h, VS-4718 (1 and 3 μM) was added 2 h prior to incubation with anticancer drugs. After 72 h of incubation, MTT solution (4 mg/ml) was added to each well and the cells were further incubated for 4 h. Then, the supernatant was discarded and 100 μL of DMSO was used to dissolve the formazan crystals. An accuSkanTM GO UV/Vis Microplate Spectrophotometer (Fisher Sci., Fair Lawn, NJ) was used to determine the absorbance at 570 nm. The concentration for 50% inhibition of cell viability (IC_50_) of the anticancer drug was calculated as previously described (Zhang et al., 2015). Verapamil (3 μM), Ko 143 (3 μM), and MK 571 (25 μM) were used to reverse ABCB1-, ABCG2- and ABCC1-mediated MDR, respectively, as positive controls. Cisplatin, known as a non-substrate of ABCB1, ABCG2, or ABCC1, was used as a negative control of anticancer drug.

### Western blotting analysis

Western blotting analysis was carried out as previously described (Zhang X. Y. et al., 2016). Briefly, cells were incubated with or without VS-4718 for varying amounts of time (0, 24, 48, 72 h) before being lysed. Protein concentrations were determined with BCA Protein Assay Kit (Pierce, Rockford, IL). Equal amounts (20 μg) of proteins were subjected to 10% sodium dodecyl sulfate polyacrylamide gel electrophoresis (SDS-PAGE) and transferred to PVDF membranes (Millipore, Billerica, MA). The presence of ABCB1 and ABCG2 was determined using monoclonal antibody F4 (dilution 1:500) and BXP-21 (dilution 1:1,000), respectively. GAPDH was used as a loading control. The resulting protein bands were analyzed using Image J software.

### Immunofluorescence assay

The immunofluorescence assay was performed as previously described (Zhang X. Y. et al., 2016). After being cultured overnight in 24-well plates, the cells (2 × 10^4^) were treated with VS-4718 for 0, 24, 48, and 72 h. Then cells were fixed in 4% paraformaldehyde for 10 min and permeabilized by 0.1% Triton X-100 for 10 min before blocking with 6% BSA for 1 h. Cells were incubated with monoclonal antibodies ABCB1 (F4, dilution 1:100) and ABCG2 (BXP-21, dilution 1:150) at 4 °C overnight. Alexa Fluor 488 conjugated secondary antibody (1:1,000) was used after washing with iced PBS. DAPI was used to counterstain the nuclei. Immunofluorescence images were collected using an EVOS FL Auto fluorescence microscope (Life Technologies Corporation, Gaithersburg, MD).

### ATPase assay

The ABCB1- and ABCG2-associated ATPase activities were measured using PREDEASY ATPase Kits (TEBU-BIO nv, Boechout, Belgium) with modified protocols. In short, cell membranes that overexpressed ABCB1 or ABCG2 were thawed and diluted before used. Sodium orthovanadate (Na_3_VO_4_) was used as an ATPase inhibitor. Various concentrations of VS-4718 were incubated with membranes for 5 min. The ATPase reactions were initiated by adding 5 mM Mg^2+^ ATP. Luminescence signals of P_i_ were initiated and measured after incubation at 37°C for 40 min with brief mixing. The changes of relative light units were determined by comparing Na_3_VO_4_-treated samples with VS-4718 treated groups.

### [^3^H]-paclitaxel and [^3^H]-mitoxantrone accumulation assay

For the [^3^H]-paclitaxel accumulation assay, KB-3-1 and its drug resistant subline KB-C2 were used. Briefly, 5 × 10^5^ cells were cultured in 24-well plates overnight before the assay, and VS-4718 was added 2 h prior to the addition of [^3^H]-paclitaxel. After incubating with [^3^H]-paclitaxel with or without VS-4718 for 2 h at 37°C, cells were washed twice with iced PBS, and lysed with 0.25% trypsin before being placed in 5 mL scintillation fluid. Radioactivity of cells was measured in the Packard TRI-CARB 1900CA liquid scintillation analyzer (Packard Instrument, Downers Grove, IL). NCI-H460, NCI-H46/MX20, were used for [^3^H]-mitoxantrone accumulation assay as previously described (Sun et al., 2012).

### [^3^H]-paclitaxel and [^3^H]-mitoxantrone efflux assay

For the efflux assay, cells were incubated with VS-4718 for 2 h followed by incubation with [^3^H]-paclitaxel or [^3^H]-mitoxantrone with or without VS-4718 for 2 h at 37°C. The cells were washed with iced PBS twice and then lysed at various time points (0, 30, 60, and 120 min) with trypsin. Subsequently, cells were placed in 5 mL of scintillation fluid, and radioactivity was measured in the Packard TRI-CARB 1900CA liquid scintillation analyzer (Packard Instrument, Downers Grove, IL). KB-3-1 and KB-C2 were used for [^3^H]-paclitaxel efflux assay. NCI-H460, NCI-H46/MX20 were used for [^3^H]-mitoxantrone efflux assay (Sun et al., 2012).

### Molecular modeling of human ABCB1 homology model and wild-type human ABCG2 model

All docking experiments were performed with software Schrodinger 2018–1 (Schrödinger, LLC, New York, NY, 2018) as described previously (Zhang Y. K. et al., 2016; Fan et al., 2018). Ligand preparation and protein preparation were essentially performed. Human ABCB1 homology model was established based on refined mouse ABCB1 (PDB ID: 4M1M), and the docking grid at drug-binding pocket was generated (Li et al., 2014). The grid of ABCG2 was generated by selecting residues at a substrate-binding pocket of ABCG2 (PDB ID: 5NJ3, selected residues: Phe432, Phe-439, Leu539, Ile543, Val546, and Met549) (Taylor et al., 2017). Glide XP docking was performed and the receptor grid for induced-fit docking (IFD) was generated by selecting the best scoring ligand. The Induced-fit docking was performed with the default protocol.

### Statistical analysis

All data are expressed as the mean ± *SD* and analyzed using one-way ANOVA. All experiments were repeated at least three times. Differences were considered significant when P < 0.05.

## Results

### The effect of VS-4718 on the efficacy of anticancer drugs in cells overexpressing ABCB1 and ABCG2 transporters

**Figure 1:**
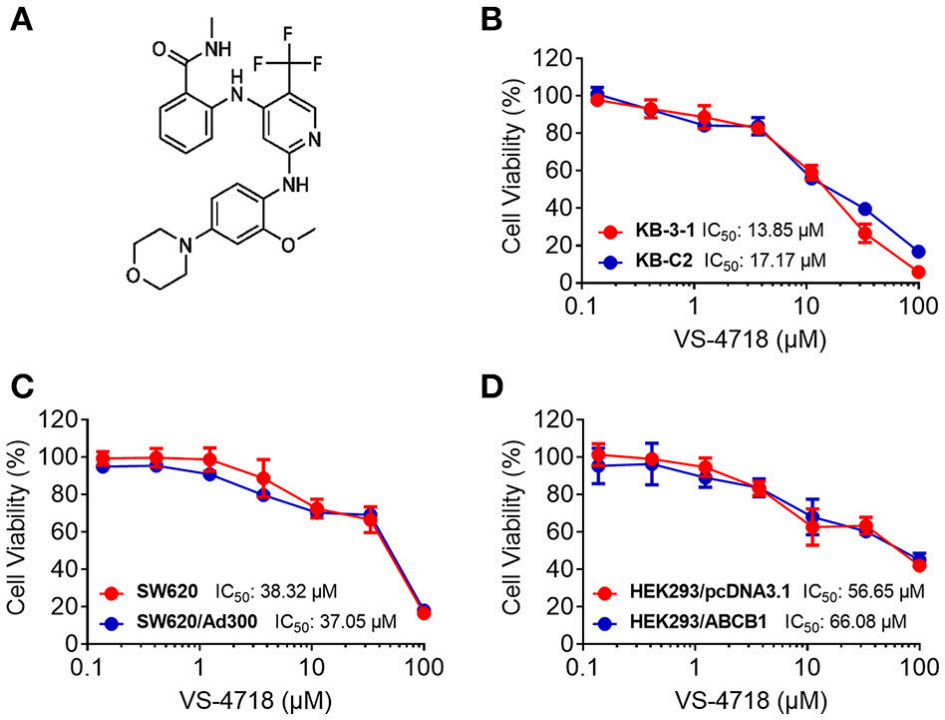
Chemical structure of VS-4718 and concentration-dependent viability curves for parental and ABCB1-overexpressing cells incubated with VS-4718. **(A)** Chemical structure of VS-4718. **(B)** Concentration-viability curves for KB-3-1 and KB-C2 cells incubated with VS-4718 for 72 h. **(C)** Concentration-viability curves for SW620 and SW620/Ad300 cells incubated with VS-4718 for 72 h. **(D)** Concentration-viability curves for HEK293/pcDNA3.1 and HEK293/ABCB1 cells incubated with VS-4718 for 72 h. The cell viability was determined by MTT assay. Data are expressed as mean ± *SD*, and representative of three independent experiments in triplicate are shown.

**Figure 2:**
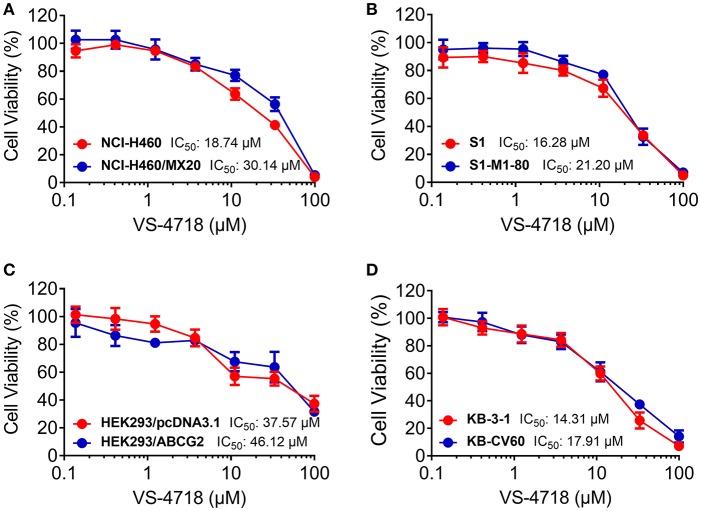
Concentration-viability curves for parental and ABCG2- and ABCC1-overexpressing cells incubated with VS-4718. **(A)** Concentration-viability curves for NCI-H460 and NCI-H460/MX20 cells incubated with VS-4718 for 72 h. **(B)** Concentration-viability curves for S1 and S1-M1-80 cells incubated with VS-4718 for 72 h. **(C)** Concentration-viability curves for HEK293/pcDNA3.1 and HEK293/ABCG2 cells incubated with VS-4718 for 72 h. **(D)** Concentration-viability curves for KB-3-1 and KB-CV60 cells incubated with VS-4718 for 72 h. The cell viability was determined by MTT assay. Data are expressed as mean ±*SD*, representative of three independent experiments in triplicate.

We first determined the toxicity of VS-4718 in the cells we would use to choose concentrations of VS-4718 that would not significantly alter cell survival rate. Concentrations of VS-4718 below IC_20_ upon 72 h-incubation with cells were selected. Based on the results (Figures 1, 2), we conducted the following assays with VS-4718 at concentrations of 1 and 3 μM.

VS-4718 significantly reduced the IC_50_ values of KB-C2 and SW620/Ad300 cells to doxorubicin and paclitaxel compared to their control cells in a dose-dependent manner (Table 1). After treatment with VS-4718, the IC_50_ values of mitoxantrone, topotecan, and SN-38 to NCI-H460/MX20 and S1-M1-80 cells were much lower than those in their control resistant cells (Table 3). Similarly, VS-4718 significantly increased the efficacy of doxorubicin and paclitaxel in the HEK293/ABCB1 cells compared with that in the control resistant cells (Table 2). Furthermore, after treatment with VS-4718, the ABCG2-transfected cells were much more sensitive to mitoxantrone, topotecan, and SN-38 compared with the control group (Table 4). VS-4718 did not alter the sensitivity of KB-CV60 to vincristine (Tables 5). In addition, VS-4718 did not significantly alter the cytotoxic effect of cisplatin, a drug that is neither a substrate of ABCB1 nor ABCG2 (Tables 1–5). These results suggested that VS-4718 could reverse ABCB1- and ABCG2-mediated MDR, but not MDR mediated by ABCC1.

**Table 1:** VS-4718 sensitized ABCB1-substrate-selected resistant cells to ABCB1 substrates.

**Treatment**	**IC_50_ ±*SD*^a^(RF^b^)**			
	**KB-3-1 (μM)**	**KB-C2 (μM)**	**SW620 (μM)**	**SW620/Ad300 (μM)**
Doxorubicin	0.035 ± 0.002 (1.00)	2.560 ± 0.250 (72.62)	0.061 ± 0.007 (1.00)	5.494 ± 0.600 (90.19)
+VS-4718 (1 μM)	0.030 ± 0.005 (0.85)	0.346 ± 0.128 (9.82)^*^	0.058 ± 0.012 (0.96)	1.120 ± 0.424 (18.39)^*^
+VS-4718 (3 μM)	0.026 ± 0.005 (0.74)	0.092 ± 0.007 (2.61)^*^	0.066 ± 0.004 (1.08)	0.382 ± 0.041 (6.27)^*^
+Verapamil (3 μM)	0.029 ± 0.007 (0.83)	0.051 ± 0.013 (1.44)^*^	0.069 ± 0.012 (1.12)	0.356 ± 0.030 (5.84)^*^
Paclitaxel	0.005 ± 0.001 (1.00)	2.348 ± 0.233 (521.83)	0.028 ± 0.003 (1.00)	2.411 ± 0.687 (86.10)
+VS-4718 (1 μM)	0.004 ± 0.001 (0.97)	0.491 ± 0.031 (109.10)^*^	0.029 ± 0.005 (1.03)	0.358 ± 0.138 (12.79)^*^
+VS-4718 (3 μM)	0.003 ± 0.001 (0.67)	0.053 ± 0.018 (11.79)^*^	0.024 ± 0.002 (0.86)	0.146 ± 0.097 (5.23)^*^
+Verapamil (3 μM)	0.004 ± 0.001 (0.89)	0.061 ± 0.011 (13.60)^*^	0.023 ± 0.002 (0.83)	0.100 ± 0.042 (3.57)^*^
Cisplatin	1.135 ± 0.038 (1.00)	1.921 ± 0.139 (1.69)	1.195 ± 0.094 (1.00)	1.228 ± 0.674 (1.00)
+VS-4718 (1 μM)	1.476 ± 0.007 (1.30)	1.862 ± 0.210 (1.64)	1.272 ± 0.347 (1.06)	1.221 ± 0.119 (0.99)
+VS-4718 (3 μM)	1.462 ± 0.022 (1.29)	2.544 ± 0.145 (2.24)	1.365 ± 0.763 (1.14)	1.490 ± 0.073 (1.21)
+Verapamil (3 μM)	1.113 ± 0.045 (0.98)	2.221 ± 0.162 (1.96)	1.374 ± 0.655 (1.15)	1.474 ± 0.169 (1.20)

a *IC_50_ values were determined by MTT assay as described in “Materials and Methods,” and were obtained from three independent experiments in triplicate*.

b *Resistance fold (RF) was calculated from dividing the IC_50_ values of resistant cells (KB-C2 and SW620/Ad300) by the IC_50_ of parental cells (KB-3-1 and SW620) in the presence or absence of VS-4718 or positive control inhibitor*.

* *indicates p < 0.05 vs. group treated with antineoplastic drug only*.

**Table 2:** VS-4718 sensitized ABCB1-gene-transfected cells to ABCB1 substrates.

**Treatment**	**IC_50_ ±*SD*^a^ (RF^b^)**	
	**HEK293/pcDNA3.1 (μM)**	**HEK293/ABCB1 (μM)**
Doxorubicin	0.065 ± 0.008 (1.00)	1.375 ± 0.079 (21.15)
+VS-4718 (1 μM)	0.066 ± 0.009 (1.01)	0.491 ± 0.049 (7.55)^*^
+VS-4718 (3 μM)	0.055 ± 0.013 (0.84)	0.220 ± 0.034 (3.39)^*^
+Verapamil (3 μM)	0.064 ± 0.007 (0.99)	0.121 ± 0.017 (1.85)^*^
Paclitaxel	0.043 ± 0.001 (1.00)	1.054 ± 0.200 (24.80)
+VS-4718 (1 μM)	0.047 ± 0.004 (1.11)	0.498 ± 0.062 (11.73)^*^
+VS-4718 (3 μM)	0.037 ± 0.005 (0.86)	0.313 ± 0.053 (7.37)^*^
+Verapamil (3 μM)	0.032 ± 0.009 (0.74)	0.297 ± 0.055 (6.99)^*^
Cisplatin	1.071 ± 0.144 (1.00)	1.259 ± 0.425 (1.18)
+VS-4718 (1 μM)	1.186 ± 0.443 (1.11)	1.260 ± 0.563 (1.18)
+VS-4718 (3 μM)	1.105 ± 0.371 (1.03)	1.272 ± 0.217 (1.19)
+Verapamil (3 μM)	0.971 ± 0.206 (0.91)	1.293 ± 0.368 (1.21)

a *IC_50_ values were determined by MTT assay as described in “Materials and Methods,” and were obtained from three independent experiments in triplicate*.

b *Resistance fold (RF) was calculated from dividing the IC_50_ values of resistance cells (HEK293/ABCB1) by the IC_50_ of parental cells (HEK293/pcDNA3.1) in the presence or absence of VS-4718 or positive control inhibitor*.

* *indicates p < 0.05 vs. group treated with antineoplastic drug only*.

**Table 3:** VS-4718 sensitized ABCG2-substrate-selected resistant cells to ABCG2 substrates.

**Treatment**	**IC_50_ ±*SD*^a^ (RF^b^)**			
	**NCI-H460 (μM)**	**NCI-H460/MX20 (μM)**	**S1 (μM)**	**S1-M1-80 (μM)**
Mitoxantrone	0.036 ± 0.008 (1.00)	4.361 ± 0.609 (120.30)	0.032 ± 0.002 (1.00)	4.559 ± 1.607 (143.23)
+VS-4718 (1 μM)	0.037 ± 0.013 (1.01)	1.247 ± 0.456 (34.40)^*^	0.029 ± 0.006 (0.91)	2.098 ± 0.916 (65.90)^*^
+VS-4718 (3 μM)	0.030 ± 0.015 (0.82)	0.496 ± 0.193 (13.68)^*^	0.027 ± 0.004 (0.85)	0.577 ± 0.182 (18.12)^*^
+Ko 143 (3 μM)	0.022 ± 0.006 (0.62)	0.302 ± 0.018 (8.32)^*^	0.026 ± 0.005 (0.81)	0.034 ± 0.015 (1.08)^*^
SN-38	0.038 ± 0.012 (1.00)	3.169 ± 0.493 (83.51)	0.043 ± 0.003 (1.00)	3.628 ± 1.819 (85.36)
+VS-4718 (1 μM)	0.031 ± 0.013 (0.82)	0.436 ± 0.287 (11.49)^*^	0.041 ± 0.009 (0.95)	0.365 ± 0.026 (8.59)^*^
+VS-4718 (3 μM)	0.030 ± 0.012 (0.78)	0.071 ± 0.015 (1.88)^*^	0.053 ± 0.006 (1.25)	0.097 ± 0.042 (2.27)^*^
+Ko 143 (3 μM)	0.031 ± 0.021 (0.81)	0.039 ± 0.003 (1.02)^*^	0.052 ± 0.013 (1.21)	0.045 ± 0.035 (1.05)^*^
Topotecan	0.067 ± 0.009 (1.00)	4.471 ± 0.644 (66.85)	0.064 ± 0.007 (1.00)	4.940 ± 0.701 (77.79)
+VS-4718 (1 μM)	0.058 ± 0.034 (0.87)	1.170 ± 0.398 (17.50)^*^	0.061 ± 0.001 (0.95)	1.801 ± 0.791 (28.36)^*^
+VS-4718 (3 μM)	0.053 ± 0.026 (0.79)	0.503 ± 0.053 (7.53)^*^	0.057 ± 0.002 (0.90)	0.434 ± 0.120 (6.84)^*^
+Ko 143 (3 μM)	0.034 ± 0.013 (0.71)	0.264 ± 0.034 (3.94)^*^	0.051 ± 0.029 (0.80)	0.306 ± 0.037 (4.81)^*^
Cisplatin	1.658 ± 0.261 (1.00)	1.696 ± 0.300 (1.02)	1.447 ± 0.064 (1.00)	1.508 ± 0.159 (1.04)
+VS-4718 (1 μM)	2.024 ± 0.421 (1.22)	1.672 ± 0.271 (1.01)	1.347 ± 0.196 (0.93)	1.873 ± 0.202 (1.29)
+VS-4718 (3 μM)	1.550 ± 1.055 (0.93)	1.699 ± 0.245 (1.02)	1.290 ± 0.051 (0.89)	1.840 ± 0.744 (1.27)
+Ko 143 (3 μM)	1.812 ± 0.801 (1.09)	1.721 ± 0.245 (1.04)	1.417 ± 0.188 (0.98)	1.506 ± 0.441 (1.04)

a *IC_50_ values were determined by MTT assay as described in “Materials and Methods,”and were obtained from three independent experiments in triplicate*.

b *Resistance fold (RF) was calculated from dividing the IC_50_ values of resistant cells (NCI-H460/MX20 and S1-M1-80) by the IC_50_ of parental cells (NCI-H460 and S1) in the presence or absence of VS-4718 or positive control inhibitor*.

* *indicates p < 0.05 vs. group treated with antineoplastic drug only*.

**Table 4:** VS-4718 sensitized ABCG2-gene-transfected cells to ABCG2 substrates.

**Treatment**	**IC_50_ ± SD^a^ (RF^b^)**	
	**HEK293/pcDNA3.1 (μM)**	**HEK293/ABCG2 (μM)**
Mitoxantrone	0.040 ± 0.004 (1.00)	0.556 ± 0.083 (13.90)
+VS-4718 (1 μM)	0.048 ± 0.009 (1.19)	0.306 ± 0.054 (7.66)^*^
+VS-4718 (3 μM)	0.033 ± 0.009 (0.81)	0.052 ± 0.004 (1.31)^*^
+Ko 143 (3 μM)	0.040 ± 0.005 (0.99)	0.036 ± 0.010 (0.89)^*^
SN-38	0.022 ± 0.002 (1.00)	0.307 ± 0.078 (13.95)
+VS-4718 (1 μM)	0.022 ± 0.002 (1.00)	0.109 ± 0.008 (4.95)^*^
+VS-4718 (3 μM)	0.019 ± 0.009 (0.85)	0.034 ± 0.001 (1.53)^*^
+Ko 143 (3 μM)	0.024 ± 0.008 (1.09)	0.024 ± 0.009 (1.09)^*^
Topotecan	0.042 ± 0.000 (1.00)	0.611 ± 0.161 (14.54)
+VS-4718 (1 μM)	0.042 ± 0.001 (0.99)	0.097 ± 0.039 (2.30)^*^
+VS-4718 (3 μM)	0.033 ± 0.005 (0.77)	0.033 ± 0.001 (0.80)^*^
+Ko 143 (3 μM)	0.041 ± 0.001 (0.96)	0.039 ± 0.006 (0.93)^*^
Cisplatin	1.088 ± 0.446 (1.00)	1.281 ± 0.206 (1.18)
+VS-4718 (1 μM)	1.009 ± 0.058 (0.93)	1.123 ± 0.057 (1.03)
+VS-4718 (3 μM)	0.840 ± 0.278 (0.77)	1.410 ± 0.230 (1.30)
+Ko 143 (3 μM)	1.014 ± 0.317 (0.93)	1.228 ± 0.526 (1.13)

a *IC_50_ values were determined by MTT assay as described in “Materials and Methods,” and were obtained from three independent experiments in triplicate*.

b *Resistance fold (RF) was calculated from dividing the IC_50_ values of resistant cells (HEK293/ABCG2) by the IC_50_ of parental cells (HEK293/pcDNA3.1) in the presence or absence of VS-4718 or positive control inhibitor*.

* *indicates p < 0.05 vs. group treated with antineoplastic drug only*.

**Table 5:** VS-4718 did not affect ABCC1-mediated MDR.

**Treatment**	**IC_50_ ±*SD*^a^ (RF^b^)**	
	**KB-3-1 (μM)**	**KB-CV60 (μM)**
Vincristine	0.012 ± 0.001 (1.00)	0.207 ± 0.011 (17.96)
+VS-4718 (1 μM)	0.013 ± 0.005 (1.09)	0.203 ± 0.005 (17.68)
+VS-4718 (3 μM)	0.013 ± 0.003 (1.09)	0.220 ± 0.035 (19.12)
+MK 571 (25 μM)	0.016 ± 0.007 (1.35)	0.092 ± 0.020 (7.98)^*^
Cisplatin	1.023 ± 0.275 (1.00)	1.261 ± 0.218 (1.23)
+VS-4718 (1 μM)	1.015 ± 0.236 (0.99)	1.381 ± 0.591 (1.35)
+VS-4718 (3 μM)	0.988 ± 0.356 (0.97)	1.344 ± 0.375 (1.31)
+MK 571 (25 μM)	1.036 ± 0.125 (1.01)	1.254 ± 0.417 (1.23)

a *IC_50_ values were determined by MTT assay as described in “Materials and Methods,” and were obtained from three independent experiments in triplicate*.

b *Resistance fold (RF) was calculated from dividing the IC_50_ values of resistant cells (KB-CV60) by the IC_50_ of parental cells (KB-3-1) in the presence or absence of VS-4718 or positive control inhibitor*.

* *indicates p < 0.05 vs. group treated with antineoplastic drug only*.

### The effect of VS-4718 on the protein expression and subcellular localization of ABCB1 and ABCG2 transporters

**Figure 3:**
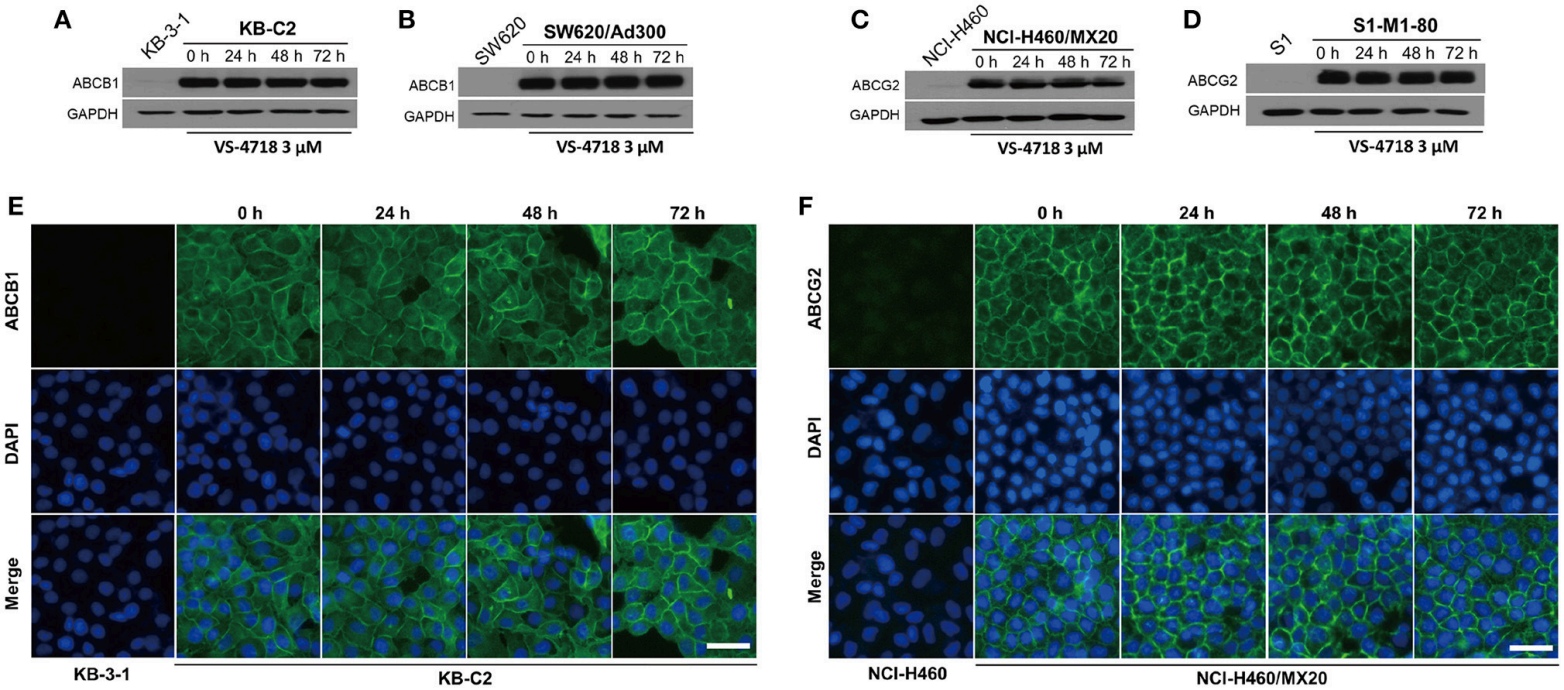
The effect of VS-4718 on the protein expression and subcellular localization of ABCB1 and ABCG2 transporters. **(A)** Detection of ABCB1 expression in KB-C2 cells incubated with 3 μM of VS-4718 for 0, 24, 48 and 72 h. **(B)** Detection and relative intensity of ABCB1 expression in SW620/Ad300 cells incubated with 3 μM of VS-4718 for 0, 24, 48, and 72 h. **(C)** Detection of ABCG2 expression in NCI-H460/MX20 cells incubated with 3 μM of VS-4718 for 0, 24, 48, and 72 h. **(D)** Detection of ABCG2 expression in S1-M1-80 cells incubated with 3 μM of VS-4718 for 0, 24, 48 and 72 h. **(E)** Sub-cellular localization of ABCB1 expression in KB-C2 cells incubated with 3 μM of VS-4718 for 0, 24, 48 and 72 h. **(F)** Data are mean ± *SD*, representative of three independent experiments. Sub-cellular localization of ABCB1 expression in NCI-H460/MX20 cells incubated with 3 μM of VS-4718 for 0, 24, 48, and 72 h. Green: ABCB1 and ABCG2. Blue: DAPI counterstains the nuclei. KB-3-1 and NCI-H460 represented the control group; Scale bar: 200 μM.

Since VS-4718 antagonized ABCB1- and ABCG2-mediated MDR, the mechanisms may result from down-regulation of the protein level and/or change of the subcellular localization of the ABC transporters. Thus, we performed Western blotting and immunofluorescence assay to determine whether VS-4718 could alter the expression level and/or the subcellular localization of ABCB1 and ABCG2 transporters. As shown in Figures 3A,B, after incubating for 24, 48, and 72 h, VS-4718 did not significantly alter the expression of ABCB1 protein (170 kDa) in ABCB1-overexpressing KB-C2 and SW620/Ad300 cells. Similarly, the expression of ABCG2 protein (72 kDa) in ABCG2-overexpressing cells NCI-H460/MX20 and S1-M1-80 was not altered significantly after treatment with VS-4718 for up to 72 h (Figures 3C,D). As shown in Figures 3E,F, ABCB1 and ABCG2 transporters were located on the membrane of KB-C2 and NCI-H460/MX20 cells separately after being treated with VS-4718 for 24 to 72 h, indicating that VS-4718 did not alter subcellular localization of the ABCB1 and ABCG2 transporters. In this study, KB-3-1 and SW620, and NCI-H460 and S1 that did not express ABCB1 and ABCG2 transporters were used as negative controls (Figure 3).

### The effect of VS-4718 on the intracellular accumulation of antineoplastic drugs in cancer cells overexpressing ABCB1 and ABCG2 transporters

**Figure 4:**
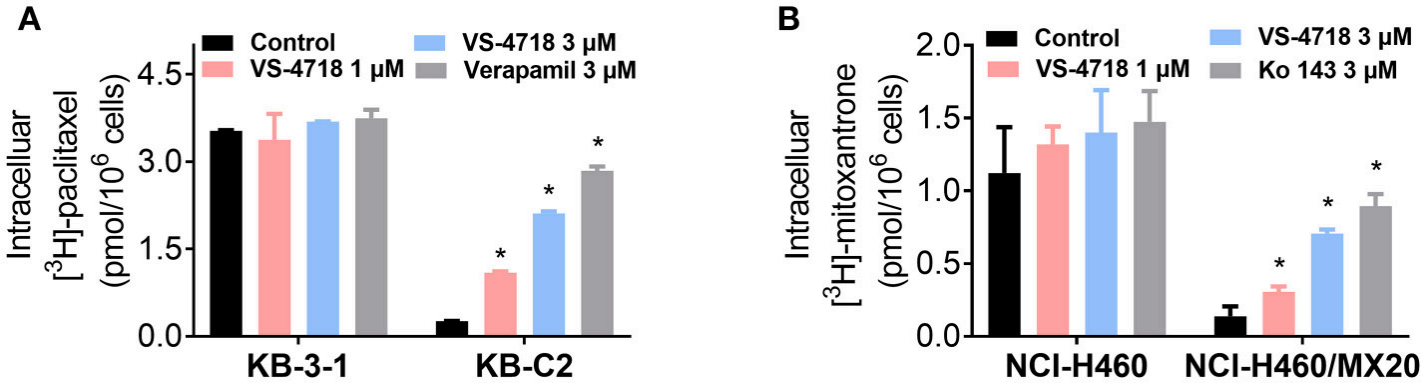
VS-4718 increased the intracellular [^3^H]-drug accumulation in cancer cells overexpressing ABCB1 and ABCG2. **(A)** The effect of VS-4718 on the accumulation of [^3^H]-paclitaxel in KB-3-1 and KB-C2 cells. **(B)** The effect of VS-4718 on the accumulation of [^3^H]-mitoxantrone in NCI-H460 and NCI-H460/MX20 cells. Verapamil (3 μM) and Ko 143 (3 μM) were used as positive controls for ABCB1 or ABCG2 overexpressing cells respectively. Data are mean, representative of three independent experiments. *p < 0.05, compared with control group.

The above results indicated that VS-4718 significantly weakened ABCB1- and ABCG2-mediated MDR. However, VS-4718 did not significantly alter ABCB1 and ABCG2 protein expression or subcellular localization. To gain insight into the mechanisms of VS-4718 in reversing MDR, an accumulation assay was performed. The intracellular levels of [^3^H]-paclitaxel and [^3^H]-mitoxantrone were measured respectively in cells that overexpress ABCB1 and ABCG2 transporters in the presence or absence of VS-4718. As shown in Figure 4A, VS-4718 significantly increased the intracellular level of [^3^H]-paclitaxel in KB-C2 cells, that overexpress ABCB1 transporters, but not in its parental cell line KB-3-1 cells. Similarly, the intracellular level of [^3^H]-mitoxantrone in ABCG2-overexpressing NCI-H460/MX20 cells significantly increased after treatment with VS-4718, compared to its parental NCI-H460 cells (Figure 4B). These results suggested that VS-4718 may increase the intracellular accumulation of antineoplastic drugs by inhibiting the function of ABCB1 and ABCG2 transporters.

### The effect of VS-4718 on the efflux activity in cancer cells overexpressing ABCB1 and ABCG2 transporters

**Figure 5:**
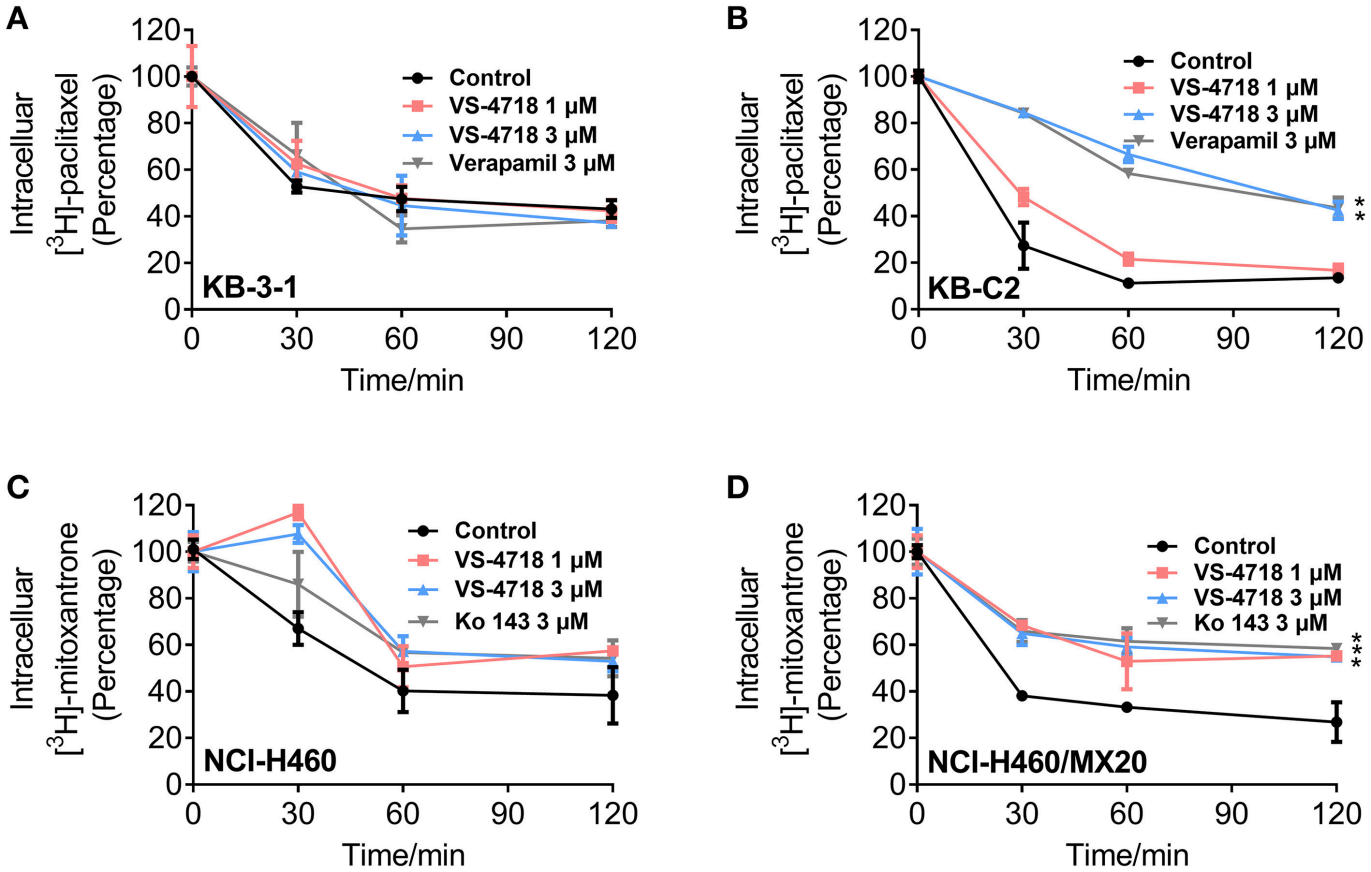
VS-4718 inhibited the efflux function of ABCB1 and ABCG2 transporters. **(A,B)** The effects of VS-4718 on efflux of [^3^H]-paclitaxel in KB-3-1 and KB-C2 cells. **(C,D)** The effects of VS-4718 on efflux of [^3^H]-mitoxantrone in NCI-H460 and NCI-H460/MX20 cells. Data are mean ±*SD*, representative of three independent experiments. *p < 0.05, compared with control group.

In order to further understand the mechanism of VS-4718 in antagonizing ABCB1- and ABCG2-mediated MDR, we performed the efflux assay to determine the effect of VS-4718 on the efflux function of ABCB1 and ABCG2 transporters. As shown in Figures 5B,D, VS-4718 significantly reduced the efflux of [^3^H]-paclitaxel in ABCB1-overexpressing KB-C2 cells, and [^3^H]-mitoxantrone efflux in ABCG2-overexpressing NCI-H460/MX20 cells. Nevertheless, VS-4718 did not significantly alter the efflux of [^3^H]-paclitaxel or [^3^H]-mitoxantrone in their parental KB-3-1 or NCI-H460 cells (Figures 5A,C). These results suggested that VS-4718 could increase the accumulation of anticancer drugs by impeding the efflux function mediated by ABCB1 and ABCG2.

### VS-4718 stimulated the ATPase activity of ABCB1 and ABCG2

**Figure 6:**
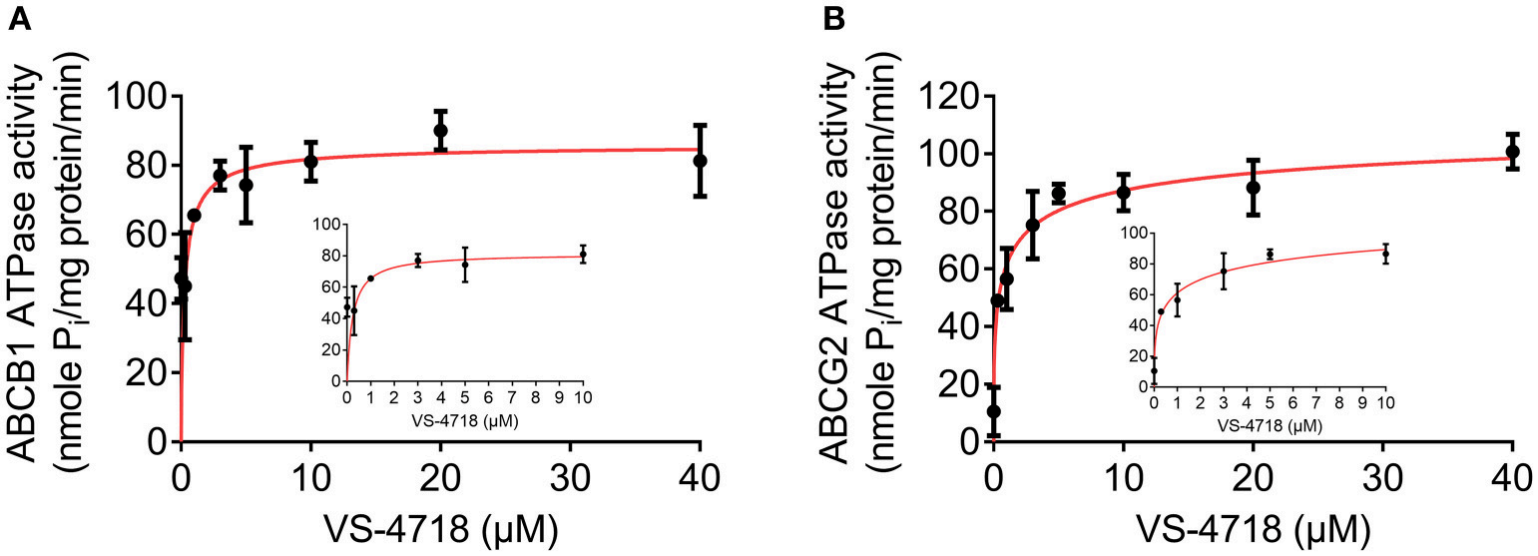
VS-4718 stimulated the ATPase activity of ABCB1 and ABCG2. **(A)** Effect of VS-4718 on the ATPase activity of ABCB1. **(B)** Effect of VS-4718 on the ATPase activity of ABCG2. The inset graphs illustrate the effect of 0–10 μM VS-4718 on the ATPase activity of ABCB1 **(A)** or ABCG2 **(B)**. Data are mean± *SD*, representative of three independent experiments.

As the above results showed that VS-4718 significantly antagonized ABCB1- and ABCG2-mediated MDR by inhibiting the efflux function of ABCB1 and ABCG2 transporters, it is likely that VS-4718 could affect the ATPase activity of ABCB1 and ABCG2 transporters. Hence, we measured ABCB1- or ABCG2-mediated ATP hydrolysis in the presence or absence of VS-4718 at various concentration from 0 to 40 μM to verify this hypothesis. As shown in Figure 6A, VS-4718 stimulated the ATPase activity of ABCB1 transporters in a dose-dependent manner with a maximal stimulation of 4.89-fold of the basal activity, and the concentration of VS-4718 required to obtain 50% of maximal stimulation is 1.72 μM. Similarly, VS-4718 stimulated the ATPase activity of ABCG2 transporters (Figure 6B), the concentration of VS-4718 required to obtain 50% of maximal stimulation is 9.60 μM, with 3.01-fold of maximum stimulation. These results suggested that VS-4718 may interact with the drug-substrate-binding site and affect the ATPase activity of ABCB1 and ABCG2 thereby restraining their efflux functions.

### Docking analysis of the binding of VS-4718 with human ABCB1 homology model and ABCG2 model

**Figure 7:**
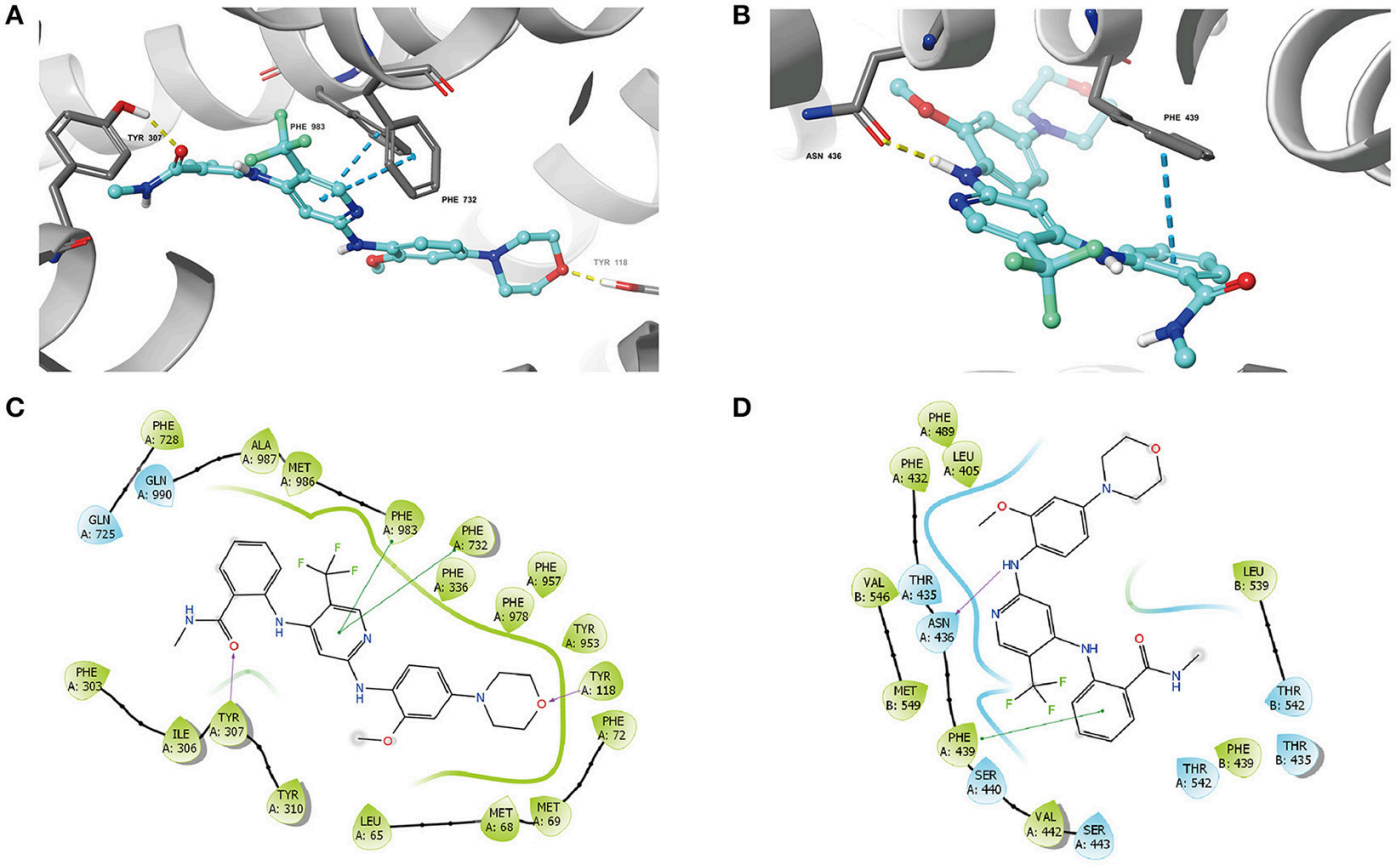
The molecular modeling study of VS-4718 with human homology ABCB1 and human ABCG2**. (A)** Docked position of VS-4718 within the drug-binding site of human ABCB1 homology model. **(B)** Docked position of VS-4718 within the binding site of ABCG2. VS-4718 is shown as ball and stick mode with the atoms colored: carbon-cyan, hydrogen-white, nitrogen-blue, oxygen-red, fluorine-green, hydrogen-white. Important residues are shown as sticks with gray color. π-π stacking interactions are indicated with cyan dotted short line. Hydrogen bonds are shown by the yellow dotted line. **(C)** The two-dimensional ligand-receptor interaction diagram of VS-4718 and human homology ABCB1. **(D)** The two-dimensional ligand-receptor interaction diagram of VS-4718 and human ABCG2. The amino acids within 3 Å are shown as colored bubbles, cyan indicates polar residues, and green indicates hydrophobic residues. The purple arrow indicates hydrogen bond and green short line shows π-π stacking interaction.

The best-scored docked position of VS-4718 within the binding pocket of human homology modeled ABCB1 and ABCG2 (5NJ3) are shown in Figure 7. The docking score of the binding of VS-4718 and ABCB1 is −10.782 kcal/mol and that of the binding of VS-4718 and human ABCG2 is −10.767 kcal/mol. There are two π-π interactions between VS-4718 and human homology ABCB1 (Figure 7A). The pyridine ring of VS-4718 has π-π interaction with both Phe732 and Phe983 of ABCB1. The oxygen in the amide group and the morpholinyl have hydrogen binding with Tyr307 and Tyr118 in chain A, respectively. Moreover, VS-4718 could be stabilized in the pocket of ABCB1 by hydrophobic interaction with residues such as Phe72, Phe303, Tyr310, Phe336, Phe953, Phe978, Met986, and Ala987 (Figure 7C). As shown in Figure 7B, the binding of VS-4718 and ABCG2 include hydrogen bonding interaction and π-π interaction. The phenyl ring in the benzamide group of VS-4718 has π-π interaction with Phe439 in the A chain. The amino group in VS-4718 as hydrogen bond (NH_2_⋯ NH_2_-Asn436) has hydrogen bonding interaction with residue Asn436 in the A chain. Besides, VS-4718 could also have hydrophilic interaction with the residues (Thr435, Asn436, Ser440, Ser443, Thr542) in the drug-binding pocket of ABCG2 (Figure 7D).

## Discussion

It is well documented that the ABC transporters expressed on cancer cell membrane are responsible for MDR, which finally leads to the failure of chemotherapy (Dassa and Bouige, 2001; Szakács et al., 2006; Shukla et al., 2008; Stavrovskaya and Stromskaya, 2008; Eckford and Sharom, 2009; Kartal-Yandim et al., 2016). In decades, studies have shown that a series of small-molecule inhibitors have the capacity to reverse ABC transporter-mediated MDR, including EGFR inhibitor gefitinib, erlotinib, AG1478, PD153035, and dacomitinib, an EGFR and HER-2 inhibitor lapatinib, a pan-HER inhibitor canertinib, a BCR-ABL inhibitor imatinib, a Bruton tyrosine kinase (BTK) inhibitor ibrutinib, and certain multi-kinase inhibitors such as sunitinib (Erlichman et al., 2001; Ozvegy-Laczka et al., 2005; Dai et al., 2008; Shukla et al., 2009; Tiwari et al., 2009; Fan et al., 2018; Zhang et al., 2018). Reversing MDR by a combination of a chemotherapeutic drug with a reversal agent against the function of ABC transporters is a potential pharmacological approach to cancer treatments.

In this study, we found that VS-4718, at non-toxic concentrations, significantly sensitized ABCB1- and ABCG2-overexpressing cancer cells to their substrates in a dose-dependent manner. However, the re-sensitizing effects were not shown in ABCC1-overexpressing cells. First of all, an MTT assay was performed to obtain non-toxic concentrations of VS-4718 in the cells that we would use to avoid possible bias of VS-4718-induced cytotoxicity in evaluating its reversal effects. Based on the MTT results, 1 and 3 μM of VS-4718 were adopted for reversal studies. Our results indicated that VS-4718 significantly increased the efficacy of doxorubicin and paclitaxel on the ABCB1-overexpressing KB-C2 and SW620/Ad300 cells compared to their control resistant cells in a dose-dependent manner. Similarly, VS-4718 significantly reduced the IC_50_ values of substrate drugs in HEK293/ABCB1 cells dose dependently. In addition, VS-4718 sensitized ABCG2-overexpressing cells, NCI-H460/MX20 and S1-MI-80, and the ABCG2-transfected HEK293 cells to mitoxantrone, topotecan, and SN-38 in a dose-dependent manner. However, VS-4718, up to 3 μM, did not significantly sensitize the parental cells such as KB-3-1, SW620, NCI-H460, S1, and HEK293/pcDNA3.1 cells. Moreover, there was no significant alteration of insensitivity of cancer cells to cisplatin, which was neither an ABCB1 nor ABCG2 substrate. Furthermore, at 1 and 3 μM, VS-4718 did not significantly alter the IC_50_ value of ABCC1-overexpressing KB-CV60 cells. These findings suggested that the reversal effect of VS-4718 is specific to ABCB1- and ABCG2-mediated MDR.

The reversal of ABC transporter-mediated MDR may due to the down-regulation of ABC protein expression or change of subcellular localization. We performed Western blotting and immunofluorescence assay to investigate the potential mechanisms. We found that there was no significant decrease in the protein level of ABCB1 or ABCG2 transporters after treatment with VS-4718 (3 μM) up to 72 h. Likewise, VS-4718 at 3 μM did not significantly change the ABCB1 and ABCG2 transporters subcellular localization after incubating for up to 72 h, suggesting that the reversal effects of VS-4718 on MDR were not related to alteration of the protein level or subcellular localization of ABC transporters. However, further studies should determine the indirect effect of VS-4718 on the expression of ABCB1 and ABCG2 at a higher concentration and a longer incubation time. Moreover, we could not exclude the possibility that part of the reversal effect of VS-4718 could involve its effect on some proteins and/or cross-talk with other proteins, which may affect the function of ABCB1 and ABCG2. This needs to be studied further to exclude this possibility. Furthermore, we could not fully exclude the possible effect of VS-4718 on ABCC1 transporter, though this kind of effect may not result in reversing ABCC1-mediated MDR, further study should be performed in the future to determine the effect of VS-4718 on protein level and/or subcellular localization of ABCC1 transporter.

In accumulation and efflux assays, we found that VS-4718 significantly increased the intracellular [^3^H]-paclitaxel concentration in ABCB1-overexpressing KB-C2 cells, and [^3^H]-mitoxantrone in ABCG2-overexpressing NCI-H460/MX20 cells, in a dose-dependent manner, while no significant [^3^H]-drug alteration was found in their corresponding parental cells. Furthermore, VS-4718 significantly prevented [^3^H]-drugs being pumped out of ABCB1- and ABCG2-overexpressing cells in a dose-dependent manner, while no significant change of efflux was observed in their corresponding parental cells. The results of the accumulation and efflux experiments were congruent with the reversal effects of VS-4718 shown in anti-cancer efficacy testing when co-administered with substrate-drugs, suggesting that VS-4718 may increase the accumulation of substrate-drugs in ABCB1- and ABCG2-overexpressing cancer cells by inhibiting the ABCB1- and ABCG2-mediated efflux activity, which led to the decline of IC_50_ of substrate-drugs and finally attenuated the ABC transporter-mediated MDR. The results are also consistent with studies of our other small-molecule reversal reagents (Zhang et al., 2017, 2018; Gupta et al., 2018).

It is known that the function of ABC transporters relies on the energy from the hydrolysis of ATP by the enzyme ATPase, which can be modulated by the presence of substrates or inhibitors (Gottesman and Ambudkar, 2001; Wilkens, 2015). Our results indicated that VS-4718 stimulated the ATPase activity of both ABCB1 and ABCG2, with a 4.89-fold in ABCB1 and a 3.01-fold in ABCG2. The results suggested that VS-4718 probably acts as a substrate of ABCB1 and ABCG2, which may competitively occupy the drug binding site of both ABCB1 and ABCG2 transporters.

Although VS-4718 presents similar up-regulated ATPase activity of ABCB1 and ABCG2, the accurate binding site still remain unclear, so molecular docking simulations of VS-4718 with substrate-binding sites of ABCB1 and ABCG2 was performed. Modeling study suggested that VS-4718 could interact with the TMD of both ABCB1 and ABCG2 with docking scores of −10.782 kcal/mol and −10.767 kcal/mol, respectively, and hydrogen bonding interactions and π-π interactions were predicted between VS-4718 and lining residues of drug-binding cavities from ABCB1 and ABCG2. In conclusion, this suggested, along with our other results, that VS-4718 acts as a potential competitive substrate that increases the ATPase activity and displaces chemotherapy drugs from ABCB1 and ABCG2 transporters, thereby inhibiting the efflux function of ABCB1 and ABCG2, and increasing the intracellular accumulation of certain substrate drugs into the MDR cells, and finally reversing MDR.

In recent years, research has shown that a series of small-molecule targeted drugs have the capacity to reverse ABC transporter-mediated MDR. However, strategies to develop ABC transporters as a therapeutic target to overcome drug resistance have, to date, failed in the clinic, due to the unpredictable fate of the co-administrated reversal reagents with anticancer drugs. Nonetheless, it has been reported that clinical resistance to chemotherapy in a series of cancers is strongly associated with the overexpression of the ABC transporters, and overexpression of ABCB1 and ABCG2 in cancers may come with poor prognosis and high risk of death (Marsh et al., 2007; Campa et al., 2012; Hlavata et al., 2012; Litviakov et al., 2013; Bartholomae et al., 2016). Our study provides a clue that the combination of VS-4718 with ABCB1 or ABCG2 substrate-drugs, like doxorubicin or topotecan, as well as SN-38, could be a novel treatment strategy to antagonize resistance in cancer patients. Furthermore, in this study, though VS-4718 (1 and 3 μM) showed relative non-toxic and significant reversal effect in the cells we used, we could not conclude that VS-4718 would work in a *in vivo* model at a non-toxic dose. Therefore, *in vivo* study should be performed in the future to support the current findings.

In conclusion, this study indicates that VS-4718 could reverse ABCB1- and ABCG2-mediated MDR by competitively inhibiting the anticancer drugs being pumped out by ABC transporters. The combination of VS-4718 with substrate drugs of ABCB1 and ABCG2 transporters might be used for cancer clinical treatment to elude MDR if it could be validated in *in vivo* models.

## Author contributions

DK and D-HY conceived the general idea. NJ, YY, C-YC, Z-NL, J-QW, PG, and Q-XT performed experiments. NJ, Z-SC, DK, and D-HY analyzed the results. NJ, YY, C-YC, Z-NL, J-QW, and PG wrote the first draft. DK and D-HY revised the manuscript.

### Conflict of interest statement

The authors declare that the research was conducted in the absence of any commercial or financial relationships that could be construed as a potential conflict of interest.
